# Expression of genes encoding the calcium signalosome in cellular and transgenic models of Huntington's disease

**DOI:** 10.3389/fnmol.2013.00042

**Published:** 2013-11-25

**Authors:** Magdalena Czeredys, Joanna Gruszczynska-Biegala, Teresa Schacht, Axel Methner, Jacek Kuznicki

**Affiliations:** ^1^Laboratory of Neurodegeneration, International Institute of Molecular and Cell BiologyWarsaw, Poland; ^2^Focus Program Translational Neuroscience, Rhine Main Neuroscience Network, Department of Neurology, Johannes Gutenberg University Medical Center MainzMainz, Germany; ^3^Laboratory of Calcium Binding Proteins, Nencki Institute of Experimental BiologyWarsaw, Poland

**Keywords:** calcium signalosome, store-operated calcium entry, transgenic mice, TaqMan low-density arrays, Huntington's disease, huntingtin, huntingtin-associated protein 1, calcyclin-binding protein

## Abstract

Huntington's disease (HD) is a hereditary neurodegenerative disease caused by the expansion of a polyglutamine stretch in the huntingtin (HTT) protein and characterized by dysregulated calcium homeostasis. We investigated whether these disturbances are correlated with changes in the mRNA level of the genes that encode proteins involved in calcium homeostasis and signaling (i.e., the calciosome). Using custom-made TaqMan low-density arrays containing probes for 96 genes, we quantified mRNA in the striatum in YAC128 mice, a model of HD, and wildtype mice. HTT mutation caused the increased expression of some components of the calcium signalosome, including calretinin, presenilin 2, and calmyrin 1, and the increased expression of genes indirectly involved in calcium homeostasis, such as huntingtin-associated protein 1 and calcyclin-binding protein. To verify these findings in a different model, we used PC12 cells with an inducible expression of mutated full-length HTT. Using single-cell imaging with Fura-2AM, we found that store-operated Ca^2+^ entry but not endoplasmic reticulum (ER) store content was changed as a result of the expression of mutant HTT. Statistically significant downregulation of the Orai calcium channel subunit 2, calmodulin, and septin 4 was detected in cells that expressed mutated HTT. Our data indicate that the dysregulation of calcium homeostasis correlates with changes in the gene expression of members of the calciosome. These changes, however, differed in the two models of HD used in this study. Our results indicate that each HD model exhibits distinct features that may only partially resemble the human disease.

## Introduction

Cellular calcium transients control a vast array of cellular functions, from short-term responses (e.g., contraction and secretion) to the long-term regulation of cell growth and proliferation. Cytosolic Ca^2+^ increases in response to activation of cell-surface receptors, either directly after the activation of ionotropic glutamate channels or indirectly after generation of the Ca^2+^-mobilizing second messenger inositol 1,4,5-trisphosphate (IP_3_). IP_3_ interacts with its receptors (IP_3_R1-3) on the membrane of the endoplasmic reticulum (ER), the major store of Ca^2+^ in the cell. The opening of these IP_3_R Ca^2+^ channels releases Ca^2+^ stored in the ER (Foskett et al., 2007). Increased cytosolic Ca^2+^ can also cause Ca^2+^-induced release of Ca^2+^ from ryanodine receptors (RyR1-3) (Hamilton, 2005). The depletion of ER Ca^2+^ stores results in activation of store-operated channels (SOCs) at the plasma membrane, mediating store-operated calcium entry (SOCE) from the extracellular space, followed by the removal of cytosolic Ca^2+^ and replenishment of luminal Ca^2+^ through sarcoplasmic/ER Ca^2+^-adenosine triphosphatases (ATPases; SERCA1-3). Considering their electrophysiological and molecular properties, two main types of SOCs can be described. The first are the highly Ca^2+^-selective calcium release activated calcium currents (ICRAC) mediated by the Orai family of proteins, which carry highly Ca^2+^-selective ICRAC currents (Vig et al., 2006). The second type of SOCs are non-selective Ca^2+^-permeable TRPC (C-type transient receptor potential) channels (Huang et al., 2006). The Ca^2+^ sensor's stromal interaction molecules 1 and 2 (STIM1 and STIM2) detect changes in Ca^2+^ concentration in the ER *via* an EF-hand Ca^2+^-binding domain, in response to store depletion, they rearrange into punctate structures that are close to the plasma membrane. This activates members of the Orai family (Orai1, Orai2, and Orai3) of Ca^2+^-influx channels (Liou et al., 2005; Roos et al., 2005), resulting in Ca^2+^ entry by ICRAC into the cell. STIM1 and Orai1 have also been implicated with cell death and mitochondrial bioenergetics (Henke et al., 2012, 2013). STIM1 and STIM2 are expressed in the brain (Klejman et al., 2009; Skibinska-Kijek et al., 2009; Ng et al., 2011; Steinbeck et al., 2011). The main function of STIM1 in neurons appears to be the activation of SOCE (Gruszczynska-Biegala et al., 2011). STIM1 directly suppresses the depolarization-induced opening of the voltage-gated Ca^2+^ channel Ca_*V*_1.2 by binding to the C-terminus of this channel, leading to the inhibition of gating and long-term internalization of the channel from the membrane (Park et al., 2010). The primary function of STIM2 in neurons was suggested to be the regulation of the resting level of Ca^2+^ in the ER and Ca^2+^ leakage (Gruszczynska-Biegala et al., 2011; Gruszczynska-Biegala and Kuznicki, 2013). To maintain the spatial and temporal properties of Ca^2+^ signals, neurons express numerous Ca^2+^-binding proteins that act as Ca^2+^ buffers (Nikoletopoulou and Tavernarakis, 2012). In the cytoplasm, parvalbumin (PV), calbindin D-28k (CALB1), and calretinin (CALB2) (Billing-Marczak and Kuznicki, 1999) act as Ca^2+^ buffers, whereas buffer proteins localized within the lumen of the ER, including calsequestrin (CASQ) and calreticulin (CALR) (Groenendyk et al., 2004; Michalak et al., 2009), allow the store to accumulate the large amounts of Ca^2+^ necessary for rapid cell signaling responses. These proteins involved in calcium signaling and homeostasis represent a special toolkit referred to as the Ca^2+^ signalosome (Berridge et al., 2003; Berridge, 2012).

Proper action of the Ca^2+^ signalosome is crucial for the functioning of neuronal cells and during aging the Ca^2+^ signaling machinery undergoes significant changes (Toescu and Verkhratsky, 2007; Puzianowska-Kuznicka and Kuznicki, 2009). Moreover, conclusive evidence indicates that neuronal Ca^2+^ signaling is abnormal in many neurodegenerative disorders (Wojda et al., 2008; Bezprozvanny, 2010). Notably, SOCE is dysregulated in epilepsy (Steinbeck et al., 2011) and AD (Jaworska et al., 2013; Ryazantseva et al., 2013). One of the neurodegenerative disorders with disrupted Ca^2+^ homeostasis is Huntington's disease (HD), which is characterized clinically by chorea, dementia, and psychiatric symptoms. This genetic disorder is caused by the expansion of a CAG trinucleotide repeat in exon 1 of the huntingtin gene (*HTT*), which is translated into polyglutamine residues (polyQ) in the huntingtin protein (HTT). Huntingtin is toxic to cells when the number of CAG repeats exceeds 36, a point at which HTT forms aggregates in the nuclei of neurons where it might inhibit the function of various proteins, including key transcriptional factors. This leads to transcriptional dysfunction and may cause neuronal degeneration (Sugars and Rubinsztein, 2003; Li and Li, 2004). HD is characterized at the molecular level by various changes, including disturbances in calcium homeostasis and signaling components, and these changes are considered to be involved in various processes that lead to the neurodegeneration observed in HD (Giacomello et al., 2011, 2013).

It is probable that destabilization of neuronal Ca^2+^ signaling is one of the toxic functions of the mutant HTT. To test this hypothesis, we used two models of HD: a cellular model and a transgenic model overexpressing mutant HTT. Inducible PC12 cells overexpress an expanded HTT with 73 glutamines (Apostol et al., 2003) and are useful for studying early changes in HD, since they accumulate aggregates of mutant HTT upon treatment with the steroid ponasterone A. Although others have used this inducible PC12 model successfully to study HD pathogenesis, no disturbances in the expression or function of the calcium signalosome have yet been described. However, it was shown that the first 17 amino-acid residues of mutated HTT modulate its sub-cellular localization, aggregation and effects on Ca^2+^ homeostasis in glutamate-challenged PC12 cells (Rockabrand et al., 2007). Our HD animal model, YAC128 transgenic mice express the full-length human HTT protein with 128 CAG repeats and display an age-dependent loss of striatal neurons, similar to those seen in human HD (Slow et al., 2003). YAC128 mice initially exhibit hyperactivity, followed by the onset of motor deficits and finally hypokinesis. Motor deficits in YAC128 mice are highly correlated with striatal neuronal loss. There is compelling evidence that supports Ca^2+^ dysfunction playing a central role in the pathogenesis of HD. In medium spiny neurons (MSNs) from YAC128 transgenic mice but not control mice, the activity of SOCE was enhanced (Wu et al., 2011). Moreover, in a yeast two-hybrid screen and YAC128 mice, an interaction between mutant HTT and the 5C-terminus of IP_3_R1 was shown to be mediated by huntingtin-associated protein-1A (Tang et al., 2003). Repeated application of glutamate to MSNs in these mice elevated cytosolic Ca^2+^ levels, resulting in apoptosis mediated by the activation of G_*q*_-coupled metabotropic glutamate receptors (Tang et al., 2005). Similar results were shown in the HdH (Q111/Q111) mouse model, in which agonist-induced Ca^2+^ release in neurons from these mice was increased relative to (Q20/Q20) control mice. Basal protein kinase B (PKB) activation was also higher in HdH (Q111/Q111) neurons, and this process was dependent on metabotropic glutamate receptor 5 (mGluR5) (Ribeiro et al., 2010).

Although the effect of mutant HTT on SOCE was previously studied in transgenic YAC128 mice (Wu et al., 2011), there is no data concerning calcium signalosome alterations, which could be considered to be involved in HD pathogenesis. Therefore, the aim of our study was to investigate whether mutated HTT is responsible for the changes in the expression of calciosome genes, and if so, which of such changes might explain the alterations observed in HD. Using custom-made TaqMan low-density arrays and individual real-time quantitative polymerase chain reaction (qRT-PCR), we studied changes in gene expression in two HD models. Our arrays contained most of the genes involved in calcium signaling and homeostasis, some genes responsible for amyloid beta precursor protein (APP) processing and insulin signaling, as well as a few genes that encode protein kinases or other proteins connected with HD. We found increased expression of few components of the calcium signalosome and genes indirectly involved in calcium homeostasis in YAC128 brains. In PC12 cells with induced expression of mutated full-length HTT, we detected downregulation of the genes that encode some proteins involved in the calcium signalosome. Our data indicate that the dysregulation of calcium homeostasis correlates with changes in the gene expression of members of the calciosome, but the two models used in this study exhibit distinct changes in gene expression.

## Materials and methods

### Animals

Female transgenic YAC128 mice that overexpressed human HTT (128 Gln) and non-transgenic littermates that were applied as controls aged between 3 and 6 months (Slow et al., 2003) were used. The animals were kept under normal laboratory conditions (12 h/12 h dark/light cycle; 50–60% relative humidity; 22°C) with food and water available *ad libitum.* Animal care was in accordance with the European Communities Council Directive (86/609/EEC). The experimental procedures were approved by the Local Commission for the Ethics of Animal Experimentation No. 1 in Warsaw (approval no. 305/212).

### Examined brain regions

The striatum (head of the caudate nucleus), the primary (M1) and secondary (M2) motor cortex, and the cerebellum were identified macroscopically according to anatomical landmarks (Paxinos and Franklin, 2004) in YAC128 and control mice brains. These brain structures were isolated and kept in −80°C until the future experiments.

### Cell cultures

PC12 cells with an inducible expression of full-length human HTT containing 73 polyglutamine repeats translated from a random codon with a TagRFP tag at the C-terminus were obtained from the Coriell Institute for Medical Research. The PC12 cells were grown to 90% confluence at 37°C in a 5% CO_2_, 96% relative humidity incubator in cell-culture plates (100 cm^2^; BD Biosciences) treated with 10 μg/μl poly-L-lysine (Sigma) in a cell culture medium that contained 15% horse serum (HS; H1270, Sigma), 2.5% fetal bovine serum (FBS; F7524, Sigma), and phenol red-free Dulbecco's Modified Eagle Medium (DMEM; 41965-039, Gibco) supplemented with 1% penicillin-streptomycin (P0781, Sigma), 0.1 mg/ml zeocin (R25001, Invitrogen), and 0.1 mg/ml geneticine (G8168, Sigma). The cells were passaged with a 1:5 dilution at least three times prior to the experiments. Each passage consisted of 5 min incubation with 2 ml of trypsin replacement (Sigma), the addition of 8 ml of cell media to inactivate the enzyme, and brief centrifugation (2 min, 300 × g), followed by media replacement. Cell suspensions were then added to the cell-culture plates and pipetted into single cells. The cells grew to a monolayer (> 50% confluence in 1–2 days), whereupon differentiation into neuron-like cells was achieved by treatment with neural growth factor (NGF 2.5S, 100 ng/ml; 13257-019, Invitrogen) for 48 h in low serum medium that contained DMEM, 1% HS, and 0.25% FBS supplemented by antibiotics as above. To induce the expression of human HTT, 2 μM ponasterone A (P3490, Sigma) was added to the differentiated cells for 48 h. As ponasterone A was dissolved in 100% ethanol, control cells were treated with the same amount of 100% ethanol in parallel with the ponasterone A-treated cells.

### Single-cell Ca^2+^ measurements

Single-cell Ca^2+^ imaging was performed using the ratiometric Ca^2+^ indicator dye Fura-2 acetoxymethyl ester (Fura-2 AM). The cells (17,000 cells/well) were differentiated into neuron-like cells in 96-well imaging plates (BD Bioscience) for 48 h and induced to express human HTT 48 h before the experiment. The cells were loaded with 5 μM Fura-2 AM (F1201, Molecular Probes) for 30 min at 37°C in Hank's Balanced Salt Solution supplemented with 2 mM CaCl_2_ and then rinsed and left undisturbed for 30 min to allow for de-esterification. Calcium-free medium instead of 2 mM CaCl_2_ contained 0.5 mM ethylene glycol tetraacetic acid (EGTA). The experiments were performed using a BD Pathway 855 High Content Imaging System (BD Biosciences). Images were acquired at 340 and 380 nm excitation wavelengths, and the ratio was calculated every 5 s for every cell.

### RNA isolation from brain tissue and PC12 cells

Brain samples were homogenized separately using a Potter-Elvehjem homogenizer. RNA was extracted using the RNeasy Lipid Tissue Mini kit with additional DNase treatment (Qiagen). To isolate RNA from PC12 cells, homogenization was performed using Qiashredders (Qiagen), and RNA was extracted using the RNeasy Plus Mini kit (Qiagen). cDNA was synthesized by reverse transcription (SuperScript III RnaseH, Invitrogen).

### Gene expression analysis

All gene expression analysis were performed using the 7900HT system (Life Technologies). Gene profiling in the brain was performed using custom-designed TaqMan low-density arrays (Life Technologies), hereinafter referred to as RT-qPCR arrays. For each array (384 assays), 1.6 μg of cDNA was loaded. The obtained data were analyzed using the relative quantification method and 2^−ΔCT^ formula (ΔCT = CT_target_ − CT_*Gapdh*_, where CT denotes the cycle threshold). The list of genes and their annotations included in the RT-qPCR arrays is shown in Table 1. For RT-qPCR arrays TaqMan chemistry (4369016, Life Technologies) was used.

**Table 1:** **List of genes and their annotations included in the RT-qPCR array**.

**Gene symbol**	**Gene name**	**Assay ID**
*Actb*	actin, beta	Mm00607939_s1
*Akt1*	thymoma viral proto-oncogene 1	Mm01331626_m1
*Akt2*	thymoma viral proto-oncogene 2	Mm02026778_g1
*Akt3*	thymoma viral proto-oncogene 3	Mm00442194_m1
*Aph1a*	anterior pharynx defective 1a homolog (*C. elegans*)	Mm03647119_g1
*Aph1b*	anteri or pharynx defective 1b homolog (*C. elegans*)	Mm00781167_s1
*Aph1c*	anterior pharynx defective 1c homolog (*C. elegans*)	Mm00503295_m1
*Apoe*	apolipoprotein E	Mm01307193_g1
*App*	amyloid beta precursor protein	Mm01344172_m1
*Atp2a1*	ATPase, Ca^2+^ transporting, cardiac muscle, fast twitch 1	Mm01275320_m1
*Atp2a2*	ATPase, Ca^2+^ transporting, cardiac muscle, slow twitch 2	Mm01201431_m1
*Atp2a3*	ATPase, Ca^2+^ transporting, ubiquitous	Mm00443897_m1
*Atp2c1*	ATPase, Ca^2+^ sequestering	Mm00723486_m1
*Bace1*	beta-site APP cleaving enzyme 1	Mm00478664_m1
*Cacna1a*	calcium channel, voltage-dependent, P/Q type, alpha 1A subunit	Mm00432190_m1
*Cacna1b*	calcium channel, voltage-dependent, N type, alpha 1B subunit	Mm01333678_m1
*Cacna1c*	calcium channel, voltage-dependent, L type, alpha 1C subunit	Mm01188822_m1
*Cacna1d*	calcium channel, voltage-dependent, L type, alpha 1D subunit	Mm01209919_m1
*Cacna1e*	calcium channel, voltage-dependent, R type, alpha 1E subunit	Mm00494444_m1
*Cacna1g*	calcium channel, voltage-dependent, T type, alpha 1G subunit	Mm00486572_m1
*Cacna1h*	calcium channel, voltage-dependent, T type, alpha 1H subunit	Mm00445382_m1
*Cacna1i*	calcium channel, voltage-dependent, alpha 1I subunit	Mm01299033_m1
*Cacybp*	calcyclin binding protein	Mm01295897_g1
*Calb1*	calbindin 1	Mm00486647_m1
*Calb2*	calbindin 2	Mm00801461_m1
*Calm1*	calmodulin 1	Mm01336281_g1
*Calr*	calreticulin	Mm00482936_m1
*Camk2a*	calcium/calmodulin-dependent protein kinase II alpha	Mm00437967_m1
*Canx*	calnexin	Mm00500330_m1
*Casq1*	calsequestrin 1	Mm00486733_m1
*Casq2*	calsequestrin 2	Mm00486742_m1
*Casr*	calcium-sensing receptor	Mm00443375_m1
*Cast*	calpastatin	Mm00807001_m1
*Cd33*	CD33 antigen	Mm00491152_m1
*Cib1*	calcium and integrin binding 1 (calmyrin)	Mm00501944_m1
*Cib2*	calcium and integrin binding family member 2	Mm00498053_m1
*Creb1*	cAMP responsive element binding protein 1	Mm00501607_m1
*Crebbp*	CREB binding protein	Mm01342452_m1
*Dbn1*	drebrin 1	Mm00517314_m1
*Fbln1*	fibulin 1	Mm00515700_m1
*Foxo1*	forkhead box O1	Mm00490672_m1
*Foxo3*	forkhead box O3	Mm01185722_m1
*Gapdh*	glyceraldehyde 3-phosphate dehydrogenase	Mm99999915_g1
*Gnaq*	guanine nucleotide binding protein, alpha q polypeptide	Mm00492381_m1
*Gria1*	glutamate receptor, ionotropic, AMPA1 (alpha 1)	Mm00433753_m1
*Gria3*	glutamate receptor, ionotropic, AMPA3 (alpha 3)	Mm00497506_m1
*Grin1*	glutamate receptor, ionotropic, NMDA1 (zeta 1)	Mm00433790_m1
*Grin2a*	glutamate receptor, ionotropic, NMDA2A (epsilon 1)	Mm00433802_m1
*Grm5*	glutamate receptor, metabotropic 5	Mm00690332_m1
*Gsk3a*	glycogen synthase kinase 3 alpha	Mm01719731_g1
*Gsk3b*	glycogen synthase kinase 3 beta	Mm00444911_m1
*Gusb*	glucuronidase, beta	Mm01197698_m1
*Hap1*	huntingtin-associated protein 1	Mm00468825_m1
*Hpca*	hippocalcin	Mm00650703_m1
*Htr3a*	5-hydroxytryptamine (serotonin) receptor 3A	Mm00442874_m1
*Htt*	huntingtin	Mm01213820_m1
*Igf1r*	insulin-like growth factor 1 receptor	Mm00802831_m1
*Insr*	insulin receptor	Mm01211875_m1
*Irs1*	insulin receptor substrate 1	Mm01278327_m1
*Irs2*	insulin receptor substrate 2	Mm03038438_m1
*Itpkb*	inositol 1,4,5-trisphosphate 3-kinase B	Mm01322781_m1
*Itpr1*	inositol 1,4,5-trisphosphate receptor 1	Mm00439907_m1
*Itpr2*	inositol 1,4,5-triphosphate receptor 2	Mm00444937_m1
*Itpr3*	inositol 1,4,5-triphosphate receptor 3	Mm01306070_m1
*Mtor*	mammalian target of rapamycin (serine/threonine kinase)	Mm00444968_m1
*Ncald*	neurocalcin delta	Mm00774745_m1
*Ncs1*	neuronal calcium sensor 1	Mm00490552_m1
*Ncstn*	nicastrin	Mm00452010_m1
*Orai1*	ORAI calcium release-activated calcium modulator 1	Mm00774349_m1
*Orai2*	ORAI calcium release-activated calcium modulator 2	Mm04214089_s1
*Orai3*	ORAI calcium release-activated calcium modulator 3	Mm01612888_m1
*Pik3ca*	phosphatidylinositol 3-kinase, catalytic, alpha polypeptide	Mm00435673_m1
*Plcb1*	phospholipase C, beta 1	Mm00479987_m1
*Plcg1*	phospholipase C, gamma 1	Mm01247293_m1
*Ppp3ca*	protein phosphatase 3, catalytic subunit, alpha isoform	Mm01317678_m1
*Psen1*	presenilin 1	Mm00501184_m1
*Psen2*	presenilin 2	Mm00448413_m1
*Psenen*	presenilin enhancer 2 homolog (*C. elegans*)	Mm00727761_s1
*Pten*	phosphatase and tensin homolog	Mm00477208_m1
*Pvalb*	parvalbumin	Mm00443100_m1
*Rcvrn*	recoverin	Mm00501325_m1
*Rgs4*	regulator of G-protein signaling 4	Mm00501389_m1
*Ryr1*	ryanodine receptor 1, skeletal muscle	Mm01175211_m1
*Ryr2*	ryanodine receptor 2, cardiac	Mm00465877_m1
*Ryr3*	ryanodine receptor 3	Mm01328421_m1
*S100a1*	S100 calcium binding protein A1	Mm01222827_m1
*S100a6*	S100 calcium binding protein A6 (calcyclin)	Mm00771682_g1
*Sept4*	septin 4	Mm00448225_m1
*Sirt1*	sirtuin 1 (silent mating type information regulation 2, homolog) 1 (*S. cerevisiae*)	Mm00490758_m1
*Slc25a3*	solute carrier family 25 (mitochondrial carrier, phosphate carrier)	Mm00728482_s1
*Stim1*	stromal interaction molecule 1	Mm00486423_m1
*Stim2*	stromal interaction molecule 2	Mm01223103_m1
*Trpc1*	transient receptor potential cation channel, subfamily C, member 1	Mm00441975_m1
*Vsnl1*	visinin-like 1	Mm01276999_m1

Single analyses of gene expression levels using cDNA preparations from PC12 cell cultures were performed in individual real-time PCR reactions, hereinafter referred to as qRT-PCR. The obtained data were quantified using the relative standard curve method. For each gene, standard cDNAs were diluted and amplified along with sample cDNAs in the same PCR run. Standard curves were generated using the 7900HT system software (Life Technologies). The quantity of mRNA in each sample (R_0_) was determined from the relative standard curve (using sample CT values) and expressed in arbitrary units corresponding to the dilution factors of the standard RNA preparation. The amplification efficiency (E) of each PCR reaction was determined using the equation of the standard curve:

$$\begin{array}{l} \text{CT}=-1/\text{log}(\text{E }+1)\times \text{log Ro }+\text{ log R}/\text{log }(\text{E +1})\\ \text{ E}=10^{-1/\text{Slope}}-1;\text{ slope}=-1/\text{log }[\text{E }+1]. \end{array}$$

Rat-specific primers were designed using Primer Blast (National Center for Biotechnology Information), and their sequences are shown in Table 2. For individual qRT-PCR in PC12 cells SYBR Green chemistry (4385612, Life Technologies) was used. The expression of the *Tmem66, Cracr2a (Efcab4b), Ryr3*, and *Atp2a1* genes was verified using FAM dye-labeled TaqMan probes (Life Technologies) and TaqMan chemistry (4369016, Life Technologies). The expression of *Cracr2a* (*Efcab4b), Ryr3*, and *Atp2a1* was undetectable in PC12 cells using both hand-made and custom-made primers.

**Table 2:** **List of primers used in individual qRT-PCR for PC12 cells**.

**PCR amplicon**	**Forward primer**	**Reverse primer**
Sept4	AGAGCATGACCCGGCTAGTA	GCCGCAGCTCTTCATCTTTC
Orai1	AGAGCATGACCCGGCTAGTA	TGCCCGGTGTTAGAGAATGG
Orai2	TCCATACTCCTGTCCTCGC	GGCCACGTGGTTGTGTTTTT
Orai3	GGTAACTATTCCCGCTGGCT	CAGCTACACCACAAACGCTG
Stim1	CTGGAGAAGAAGCTGCGTGA	TTTTGGCGGCTCCTCTCATT
Stim2	TGTCTTTGCCATGGCTGGAT	CTTCTGTGGGCACACTCCAT
Ryr1	GGACTACCTGTACATGGCTTAC	CCTCTTCTTCACCTCCTTCTTC
Ryr2	CTCCTCACCTGGAAAGGATAAG	GTCATCTCTAACCGGACCATAC
Atp2c1	TCATTCGAAAACCCCCTCGG	GAAGCTCTCGCCAGAAGACA
Atp2a3	CACAGTAGCCCGGAGGAGAA	TGTCACCGAGAAGCGACG
Atp2a2	TCACACAAAGACCGTGGAGG	CTTCTTCAGCCGGCAATTCG
Itrp1	TCTGGAAAGCTGCTAAGCCC	ATGACCGTCCCCAGCAATTT
Itpr2	GAGTCCAACCTCTTGAGCCC	TCCGGTAGTTGTTGCCCTTG
Itpr3	CGTCATGAACCACGGACTGA	ACTCGTCTTTGGAGGGCTTG
Trpc1	AAGGCTGCTTTCCGTTCACT	TACATCTCAAGCCGCAAGCA
Trpc3	ATACCTTCACCATGCGGAGC	TCACTGCTTGGAGTGCTGAG
Trpc5	AGCAGCACTCTATGTGGCAG	GCACCCCGGATTTCACCTAA
Calm1a	TGTCAGCAGCCAGTTTACC	ACCCGTTTCCTGCACATCAT
Calm2	AAGTGTGGAGTTGTGAGCGT	ACGAGTGAGTACCGGACAGA
Calm3	TGCCCGTTCTCCTGATCTCT	GCGTTTGCTAGAACCGGGTA
Calm4	GTGTTCCGGGTCTTTGACCA	CATTCAGCTCCTCCTCGGAC
Post	GTGCTAGCTGCGATGACTCT	GATGGTTTCAGGAAGGCCGA
Golli	AGCATCTGAGAAGGCCAGTAA	ATCTGCCTCCCCAAACACATC
Gapdh	TGACTCTACCCACGGCAAGTTCAA	ACGACATACTCAGCACCAGCATCA

### Immunoblotting

Cells were extracted in ice-cold RIPA buffer: 50 mM Tris, pH 7.5, 150 mM NaCl, 1% NP-40, 0.5% NaDOC, 0.1% sodium dodecyl sulfate (SDS), and 1 mM ethylenediaminetetraacetic acid (EDTA) that contained mini complete protease inhibitor cocktail (Roche) and phosphatase inhibitors (Sigma). The lysed cells were centrifuged at 16,000 × g for 30 min at 4°C. After measuring the protein concentrations with the BC Assay Protein Quantification Kit (Interchim), 100 μg protein was loaded on 3–8% Tris-acetate gel (Invitrogen). The proteins were transferred to a nitrocellulose membrane using the iBlot system (Invitrogen), fixed with Ponceau S solution (Sigma) for 20 s, and then blocked for 1 h with 3% dry non-fat milk in phosphate-buffered saline with 0.01% Tween-20 (PBS-T) at room temperature under gentle shaking. The membranes were incubated overnight at 4°C against actin (MAB1501, 1:4000, Millipore) and huntingtin (1756-1, 1:1000, Epitomics) diluted in PBS-T that contained 3% dry non-fat milk. After washing three times with PBS-T, the membranes were incubated with the secondary antibody IRDye 800 goat anti-rabbit immunoglobulin G (IgG) and IRDye 680 goat anti-mouse IgG, (both from Licor, 926-32211 and 926-32220, respectively) for 1 h at room temperature. The secondary antibodies were diluted to 1:30000 in 3% dry non-fat milk in PBS-T. Afterward, the membranes were washed three times with PBS-T and scanned for infrared fluorescence at 680 or 800 nm using an Odyssey scanner system (Licor). Brain tissue was homogenized on ice using a glass-homogenizer (70 strokes) and cleared by centrifugation at 12,000 × g for 20 min. Protein extracts (20 μg) were separated on 10% SDS-polyacrylamide gel electrophoresis (PAGE), transferred to a Protran nitrocellulose membrane (Whatman), and blocked for 2 h at room temperature in TBS-T: 50 mM Tris-HCl, pH 7.5, 150 mM NaCl, and 0.1% Tween 20 plus 5% dry non-fat milk. Nitrocellulose sheets were then incubated at 4°C overnight in blocking solution with primary monoclonal antibodies against HAP1 (611302, DB Transduction Laboratories) diluted to 1:300; CacyBP/SIP (ab51288, Abcam), 1:1000; Cib2 (H00010518-A01, Abnova), 1:100; pan-cadherin (ab6528, Abcam), 1:2500; as well as with primary polyclonal antibodies against CIB1 (11823-1-AP, Proteintech Europe), 1:100; CALB2 (7699/3H, Swant), 1:500; and glyceraldehyde 3-phosphate dehydrogenase (sc-25778, GAPDH) (Santa Cruz), 1:1000. The appropriate horseradish peroxidase-conjugated secondary antibody anti-mouse IgG (A9044) and anti-rabbit IgG (A0545), both from Sigma, was added at a dilution of 1:10000 for 1 h at room temperature. The peroxidase was detected with enhanced chemiluminescence (Amersham Biosciences). The intensity of the bands was measured using an ImageQuant LAS 4000 (GE Healthcare) and Quantity One software (Bio-Rad). Densitometry was then performed using the intensity of GAPDH or pan-cadherin bands as an internal standard depending on the molecular weight of the analyzed protein.

### Statistical analysis

**Figure 1:**
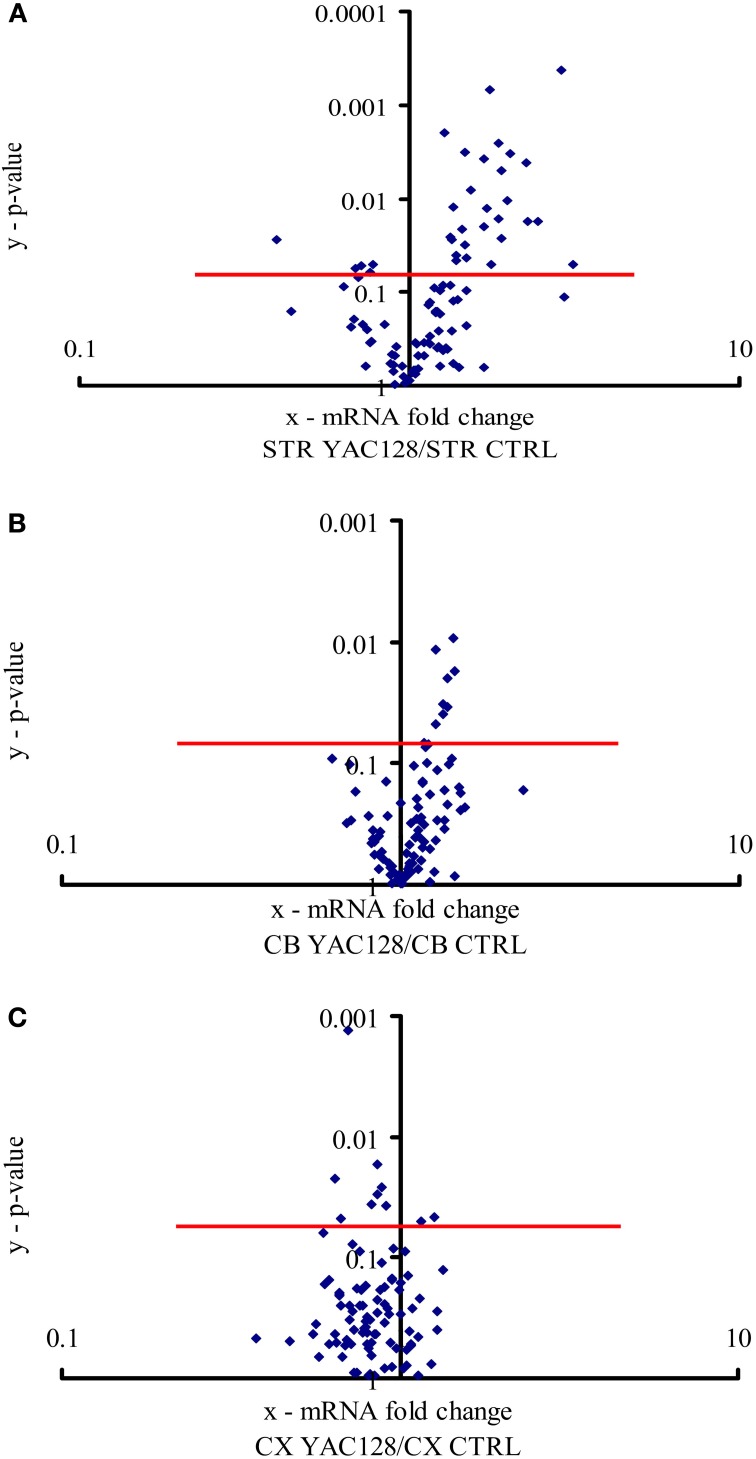
**Expression of calcium signaling and homeostasis genes in the striatum, cerebellum and motor cortex**. The volcano plot arranges genes along the dimensions of (*x*) mean expression fold difference between the analyzed brain structures: **(A)** striatum (STR), **(B)** cerebellum (CB), and **(C)** motor cortex (CX) in YAC128 mice and control mice (CTRL), and (*y*) *p*-value (Student's *t*-test). A logarithmic scale is used. Points located above the red lines represent genes whose expression was significantly changed (*p* < 0.05). The results represent data based on three independent mRNA preparations from the studied brain structures of 3-month-old mice.

To calculate *p*-values for differences in gene expression levels between the same brain regions (striatum or motor cortex or cerebellum) in wildtype and transgenic mice, we applied two-tailed paired Student's *t*-test using ΔCT values for the results obtained with the RT-qPCR arrays (Figure 1; *n* = 3) or relative values for the results obtained with individual qRT-PCRs (Table 4; *n* = 3). The degree of significance *vs*. control is indicated by asterisks: ^*^p < 0.05, ^**^p < 0.005, ^***^p < 0.0005 (ns, not significant, *p* > 0.05). To make the criteria for statistical significance more stringent, the Bonferroni correction (*p* < 0.00052) was applied. This was calculated as the *p*-value (0.05) divided by 96, which is the number of studied genes (Noble, 2009).

## Results

### Differences in gene expression between transgenic HD mice (YAC128) and wildtype mice in the striatum, cerebellum and motor cortex

To estimate the relative mRNA levels of the ensemble of genes that encode the calciosome, custom-made RT-qPCR arrays were prepared containing genes expressed in the mouse brain according to the Allen Brain Atlas (www.brain-map.org; accessed July 2, 2013; Table 1). We dissected the striatum from 3-month-old YAC128 mice and non-transgenic controls and analyzed the expression of four standards, 57 calciosome genes, as well as genes implicated in HD and Alzheimer's disease (AD) using two technical replicates. For comparison, we also performed these analyses using preparations of the cerebellum and motor cortex.

All 96 genes present in our custom-made PCR arrays were detected in the forebrain. In the striatum, ~31 of the analyzed genes exhibited a statistically significant difference (*p* < 0.05) in expression between YAC128 mice and control mice (Figure 1A). Twenty-seven genes were expressed at a higher level in HD transgenic mice compared with non-transgenic mice, and four genes were expressed at a lower level. Statistically significant differences between gene expression in YAC128 mice and control mice were observed in the cerebellum and motor cortex. In the cerebellum, eight genes exhibited enhanced expression in HD transgenic mice, and in the cortex, two had enhanced expression and eight reduced expression relative to control animals (Figures 1B,C).

### Expression of genes potentially implicated in HD in YAC128 mice

To identify genes with potential significance in this HD animal model, we focused on genes whose expression was changed at least 1.5-fold in the striatum; namely, *Hap1* (huntingtin-associated protein 1), *Calb2* (calretinin), *Aph1* (anterior pharynx defective 1 homolog) isoforms a, b and c, *Psenen* (presenilin enhancer 2 homolog), *Psen2* (presenilin 2), *Cib1* (calmyrin 1), *CacyBP/SIP* (calcyclin-binding protein), *Calr* (calreticulin), and *Cib2* (calmyrin 2).

The expression of *Hap1, Calb2*, and *Aph1b* was approximately three-times higher in the striatum of HD mice compared to control mice. When we employed the Bonferroni correction, of the 31 genes whose mRNA levels were significantly changed in the striatum in YAC128 mice, only the *Aph1b* gene met the criterion of *p* < 0.00052.

The most prominent changes in gene expression between 3-month-old YAC128 mice and non-transgenic mice were observed in the striatum (Figure 1A). This is consistent with the fact that the first changes that occur in the brains of patients suffering from HD also appear in the striatum (Vonsattel et al., 1985). Since the clinical and pathological changes become more apparent with age, we analyzed the same brain structure in 6-month-old YAC128 mice and wildtype animals. Equal amounts of cDNA obtained from the striatum in three transgenic HD mice and three control mice were prepared, and real-time PCR was performed using our RT-qPCR array. The most significant changes that were observed in the striatum in 3-month-old YAC128 mice were also observed in 6-month-old HD animals. The genes whose expression exhibited at least a 1.3-fold increase in 6-month-old mice were compared to the list of genes that were overexpressed in the striatum in 3-month-old mice. Eleven genes were identified that had significantly higher expression in the striatum in mice YAC128 of both ages (Table 3) comparing to control mice.

**Table 3:** **Gene expression analysis in the striatum in YAC128 mice**.

**Gene symbol**	**Gene name**	**Striatum (3 months)**		**Striatum (6 months)**
		**Student's *t*-test**	**Relative quantification**	**Relative quantification**
*Hap1*	huntingtin-associated protein 1	*	3.1	1.8
*Calb2*	calbindin 2 (calretinin)	ns	2.9	2.1
*Aph1b*	anterior pharynx defective 1b homolog (*C. elegans*)	***	2.9	1.9
*Psenen*	presenilin enhancer 2 homolog (*C. elegans*)	*	2.3	1.5
*Aph1a*	anterior pharynx defective 1a homolog (*C. elegans*)	**	2.3	1.9
*Psen2*	presenilin 2	*	2.0	1.6
*Cib1*	calcium and integrin binding 1 (calmyrin 1)	**	2.0	1.3
*CacyBP/SIP*	calcyclin binding protein	*	1.8	1.6
*Aph1c*	anterior pharynx defective 1c homolog (*C. elegans*)	*	1.7	1.9
*Calr*	calreticulin	*	1.5	1.6
*Cib2*	calcium and integrin binding family member 2	**	1.5	1.8
*Bace1*	beta-site APP cleaving enzyme 1	*	0.7	0.8
*Rgs4*	regulator of G-protein signaling 4	*	0.8	0.9

The striatum from 3- and 6-month-old YAC128 mice was compared to age-matched control mice using custom-made TaqMan low-density PCR arrays. Only statistically significant gene expression changes are shown:

* p < 0.05,

** p < 0.005,

*** P < 0.0005, with the exception of Calb2. Cut-offs of relative quantification (RQ) > 1.3 for upregulated genes and RQ < 0.75 for downregulated genes were applied as criteria for differential expression in transgenic mice, with the exception of Rgs4, which did not meet the additional criterion RQ < 0.75. The results for control mice were normalized to a value of 1. The gene expression results were normalized to GAPDH (n = 3 independent biological samples for 3-month-old YAC128 and age-matched control mice). The final concentration of cDNA for the RT-qPCR array was 1.6 μg. This amount contained the combination of equal amounts of cDNA obtained either from the striatum of three 6-month-old transgenic HD mice or from three age-matched control mice.

In addition to the genes that had higher expression in the striatum in YAC128 mice, two genes were downregulated in both age cohorts: *Bace1*, a gene that encodes the β-secretase component (β-site APP cleaving enzyme 1), and *Rgs4*, a member of the family of guanosine triphosphatase (GTPase)-activating proteins that regulate G_*q*_ and G_*i*_ proteins (Huang et al., 1997). However, in contrast to the up to three-fold increases associated with the other genes, the level of these two mRNAs decreased by only 10–30% (0.7–0.9-fold).

In the group of genes presented in Table 3, only *Aph1b* had *p* < 0.00052, as calculated using the Bonferroni correction. However, we also identified 12 other genes that did not meet the Bonferroni correction criterion. Nevertheless, for these genes, we observed high relative quantification and the same extent of mRNA changes in the striatum in both HD mice groups compared with control mice. We therefore chose to check some of these results at the protein level.

### Verification of gene expression data at the protein level determined by western blot

**Figure 2:**
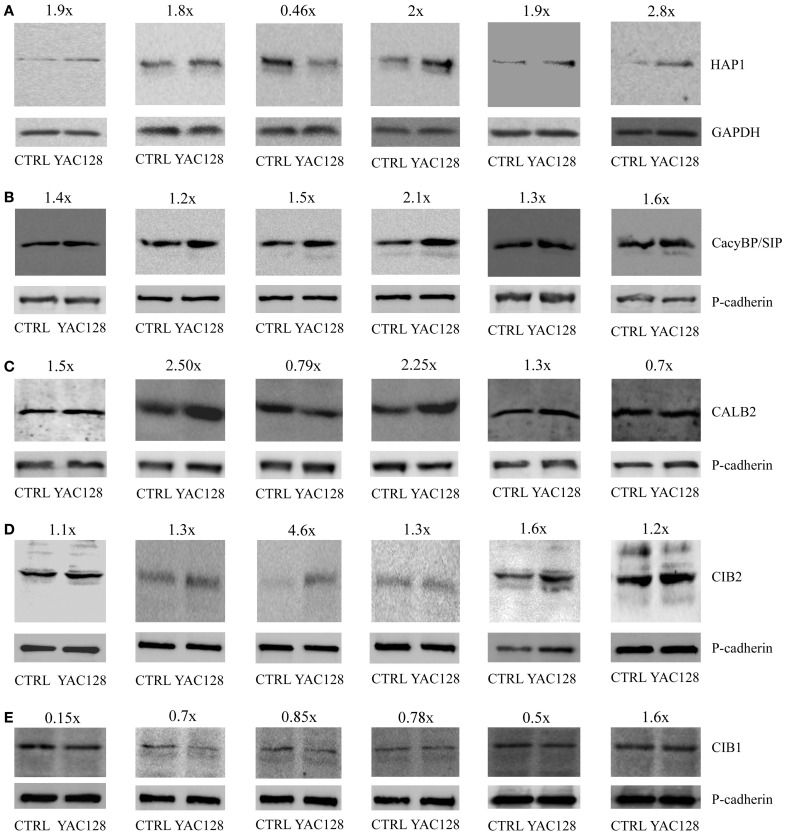
**Protein expression analysis in the striatum in YAC128 mice**. Immunoblots of HAP1 **(A)**, CacyBP/SIP **(B)**, CALB2 **(C)**, CIB2 **(D)**, and CIB1 **(E)** in the striatum of YAC128 mice and age-matched control mice (CTRL) are shown. First two blots show the data from 3- month-old mice, third and fourth blots—from 4-month-old mice, and fifth and sixth blots from 6-month-old animals. 20 μg of protein was loaded on the gel. HAP1 densitometry was performed using the intensity of GAPDH **(A)**. Pan-cadherin bands were used as an internal standard for CacyBP/SIP **(B)**, CALB2 **(C)**, CIB2 **(D)**, and CIB1 **(E)**. The fold change of the studied proteins is shown above the immunoblots.

Protein extracts from the striatum of 3-, 4-, and 6-month-old YAC128 and age-matched control mice were prepared, separated by SDS-PAGE, and incubated with primary antibodies against HAP1, CacyBP/SIP, CALB2, CIB2, CIB1 and GAPDH or pan-cadherin as loading controls. In six independent western blot experiments, we observed increase in CacyBP/SIP and CIB2 proteins in the striatum of YAC128 mice compared to control mice (Figures 2B,D). In five out of six blots there was an increase in HAP1 protein (Figure 2A) and a decrease in CIB1 (Figure 2E). In four out of six blots we observed increase in CALB2 (Figure 2C). This confirms that screening for changes at the mRNA level is feasible but must be followed by verification at the protein level.

### Reduction of SOCE by expression of mutated huntingtin in PC12 cells

**Figure 3:**
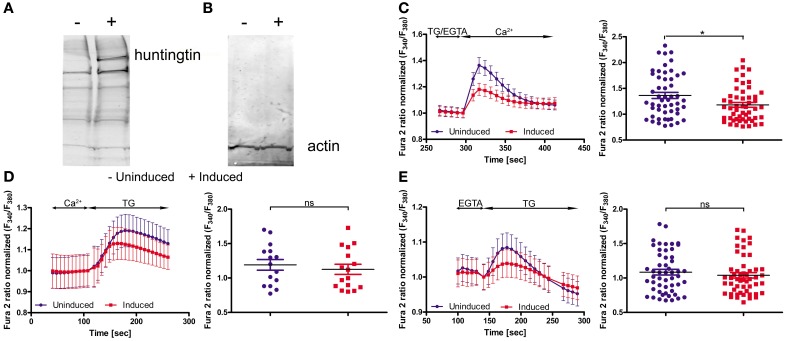
**Huntingtin expression in PC12 cells reduces SOCE**. Immunoblots of huntingtin **(A)** and actin **(B)** in induced (+) and uninduced (−) PC12 cells are shown. Ratiometric Fura-2 analysis of uninduced and induced PC12 cells was performed on a BD Pathway high-content imaging system. **(C)** SOCE measurements began in a buffer supplemented with 0.5 mM EGTA, which was then replaced by a buffer with 0.5 mM EGTA and 2 μM thapsigargin (TG). After 2.5 min, the readdition of 2 mM Ca^2+^ to the extracellular media resulted in Ca^2+^ influx. The traces show only Ca^2+^ readdition after store depletion. F_340_/F_380_ values beginning just before the readdition of Ca^2+^ were normalized to the same values (1). The ER calcium stores were depleted by the addition of 2 μM TG in the presence **(D)** or absence **(E)** of extracellular Ca^2+^. F_340_/F_380_ values beginning just before the addition of TG were normalized to the same values (1) (**C–E**, left panels). The data from different experiments were averaged (**C–E**, right panels). Summary data present the maximum (peak) of the F_340_/F_380_ ratio after the addition of Ca^2+^ or TG are expressed as the mean ± SD of the data shown in the left panels. ^*^p < 0.05; ns, not significant (*p* > 0.05). Averaged traces from **(C)** 52 individually measured wells that contained a total of ~2,000 cells per trace, **(D)** 52 individually measured wells that contained a total of ~2,000 cells per trace, and **(E)** 16 individually measured wells that contained a total of ~500 cells per trace.

To functionally evaluate our results at the cellular level, we adopted a cellular model of HD; namely, inducible PC12 cells (Apostol et al., 2003), in which an expanded HTT with 73 glutamines is expressed upon treatment with the insect steroid ponasterone A (Figure 3A). We first investigated the effects of mutated HTT on intracellular Ca^2+^ homeostasis and the expression of the genes identified in the first part of the study. Cultured PC12 cells were separated into two portions; one was treated with ponasterone A for 48 h to induce the expression of mutated human HTT and the other one with vehicle. For the calcium imaging experiments, both cultures were incubated with Fura-2AM. SOCE was examined by incubating the cells in a calcium-free medium (0.5 mM EGTA) followed by store depletion with 2 μM of the SERCA pump inhibitor thapsigargin (TG). The reintroduction of extracellular Ca^2+^ evoked a robust Ca^2+^ influx that corresponded to SOCE in both types of PC12 cells. However, cells with human HTT exhibited a significant reduction in Ca^2+^ reentry compared with control cells (Figure 3C).

In order to check if mutant HTT affect the ER Ca^2+^ store content in PC12 cells, we incubated cells either in the absence of extracellular Ca^2+^ or in the presence of 2 mM Ca^2+^. Next, 2 μM TG was added to inhibit Ca^2+^-ATPase and the amount of calcium ions released from ER were detected. The TG-releasable pool was smaller under both conditions in the cells with induced expression of HTT, but the difference did not reach statistical significance (Figures 3D,E). PC12 cells expressing mutant HTT exhibited significant differences in SOCE, which is in line with our hypothesis that HD is caused or accompanied by changes in the calciosome.

Next, we tried to verify the expression of those calciosome components that were regulated in YAC128 mice using induced PC12 cells. Surprisingly, we did not detect changes in *Hap1, Calb2, Aph1b*, or *CacyBP/SIP* gene expression in this model. We then chose genes that encode proteins known to be directly involved in SOCE or genes that encoded proteins that regulate SOCE activity to find a possible explanation for the reduced SOCE in inducible huntingtin-expressing PC12 cells. As shown in Table 4, we detected a statistically significant downregulation of Orai calcium release-activated calcium modulator 2 (*Orai2*), calmodulin 3 (*Calm*3), and septin 4 (*Sept4*). No changes in other genes that encode proteins that directly participate in SOCE, such as STIMs, RYRs, IP_3_Rs, SERCAs, and other Orais, were detected. No changes were found in other genes that encode proteins that indirectly regulate SOCE, such as TRPCs, CALMs, Golli, Post, and TMEM66. These data suggest that different mechanisms contribute to changes in calcium homeostasis in inducible huntingtin-expressing PC12 cells and transgenic YAC128 mice.

**Table 4:** **Gene expression analysis of induced and uninduced PC12 cells**.

**Gene symbol**	**Gene name**	**Student's *t*-test**	**Relative quantification**
*Orai2*	Orai calcium release-activated calcium modulator 2	*	0.8
*Calm3*	calmodulin 3	*	0.8
*Sept4*	septin 4	*	0.8

PC12 cells induced by ponasterone A were compared to uninduced cells using qRT-PCR. The gene expression results were normalized to GAPDH. Data from uninduced PC12 cells were normalized to a value of 1. Only statistically significant gene expression changes are shown (

* p < 0.05). The results represent data based on three independent mRNA preparations.

## Discussion

Several studies have investigated gene expression in HD models and HD patients using microarray analysis and discovered genes whose expression is altered (Luthi-Carter et al., 2000, 2002a,b; Desplats et al., 2006; Hodges et al., 2006). We expanded these studies to the YAC128 transgenic mice because they exhibit a slow, long-term progression of symptoms characteristic of HD (Slow et al., 2003), which may help identify the primary effects of mutant HTT. We focused on genes that encode members of calcium signalosomes (calciosome) because changes in calcium signaling and homeostasis components have been suggested to be early steps in the development of HD (Giacomello et al., 2011, 2013).

We found that ~32% of the analyzed genes in the striatum exhibited statistically significant changes in expression in YAC128 mice compared with control mice (Figure 1A). In the cerebellum and motor cortex, the levels of expression of fewer genes differed between YAC128 mice and wild type mice (Figures 1B,C), which is consistent with changes occurring first in the striatum of patients suffering from HD (Vonsattel et al., 1985). In the striatum, one gene, *Aph1b*, was positively regulated, even after a Bonferroni correction (*p* < 0.00052) to correct for multiple testing. Nevertheless, ~13% of genes in the striatum met the arbitrary criteria after cut-offs of relative quantification results (RQ > 1.3 for upregulated genes and RQ < 0.75 for downregulated genes) were applied (Table 3). They were characterized by the same extent of mRNA changes in the striatum in both groups, 3- and 6-month-old HD mice, as compared with control mice. Western blots were performed to check, if the change of specific mRNA level affect the level of a particular protein (Figure 2).

At the top of our list, presenting the highest score for relative quantification in the striatum in transgenic YAC128 mice compared with control mice, is the mRNA coding for huntingtin-associated protein 1 (Table 3). Our results revealed a three-fold increase in the expression of *Hap1* in the striatum in YAC128 mice compared with control mice. This is consistent with data obtained using another transgenic mouse model, R6/2. The enrichment of several mRNAs, including *Hap1* (although not statistically significant), was observed in neuronal nitric-oxide-synthase-positive interneurons compared with medium spiny projection neurons (Zucker et al., 2005).

*Hap1* did not meet the criterion of the Bonferroni correction (*p* < 0.00052), however, an increase in HAP1 protein was observed in brain extracts from five out of six studied YAC128 mice using western blotting (Figure 2A). In these five samples about 1.8–2.8-fold increase at the protein level of HAP1 was observed, except one sample, which exhibited 0.46-fold decrease of HAP1. Also, this sample showed unusually high level of CIB2 protein as compared to other five samples (Figure 2D). HAP1 is known to play a role in signal transduction, the regulation of vesicular transport, gene transcription, and the regulation of membrane receptor recycling (Wu and Zhou, 2009). It was identified as an HTT-interacting protein in yeast two-hybrid screens (Li et al., 1995). HAP1 protein binds more tightly to HTT with an expanded glutamine repeat than to wildtype HTT, and the binding is enhanced by lengthening the glutamine repeat (Li et al., 1998). A IP_3_R1-HAP1A-HTT ternary complex was identified in the brain that facilitates IP_3_R1-mediated intracellular Ca^2+^ release in MSNs and is activated only by mutant HTT (Tang et al., 2003). Interactions between HTT and the IP_3_R1 C-terminus depend on both, the presence of HAP1 and polyglutamine expansion. Mutant HTT can bind to the IP_3_R1 C-terminus either directly or indirectly through HAP1. The functional effects of mutant HTT on IP_3_R1-mediated Ca^2+^ release are attenuated in medium spiny striatal neurons in *Hap1* knockout mice compared with MSNs in wildtype mice (Tang et al., 2004).

Members of calcium signalosomes, which were significantly upregulated in the striatum in YAC128 mice, were *Calb2, CacyBP/SIP, Cib1, Cib2*, and *Carl*. An increase at the protein level in samples from six animals was observed for CacyBP/SIP protein and CIB2 (Figures 2B,D). CALB2 protein increased in four samples (1.5, 2.5, 2.25, and 1.3-fold), and two were characterized by a slightly lower expression of CALB2 in the striatum (0.79 and 0.7-fold). Taking into account the possible fluctuations in neuronal calcium, this result can be explained by the fact that some antibodies to calcium-binding proteins, including CALB2, preferentially recognize particular calcium-induced protein conformations (Winsky and Kuznicki, 1996). CALB2 is a member of the EF-hand family of calcium-binding proteins, which interacts with wildtype HTT but preferentially with mutant HTT (Dong et al., 2012). In neuronal cell models of HD, the overexpression of CALB2 reduced mutant HTT-induced cytotoxicity, whereas knockdown of *Calb2* enhanced mutant HTT-induced neuronal cell death (Dong et al., 2012). It was suggested that CALB2 may protect only medium-sized neurons against neurodegeneration in HD (Cicchetti and Parent, 1996). However, neurodegenerative processes that play a role in HD led to a decrease in the density of CALB2 in large striatal interneurons, without causing their death (Massouh et al., 2008). An increase in the expression of *Calb2* in the striatum in YAC128 mice may indicate the presence of a defense mechanism against mutant HTT-induced cytotoxicity. However, high-throughput analysis did not identify an increase in *Calb2* in HD patients (Hodges et al., 2006). In contrast, a decrease in the expression of *Calb1*, which encodes another 6 EF-hands calcium binding protein, calbindin, was found in *post-mortem* brain samples from the caudate of HD patients (Hodges et al., 2006). This is consistent with the substantial loss of neurons in the neostriatum that contain calbindin, suggesting that a failure of calcium buffering may contribute to cell death in HD (Seto-Ohshima et al., 1988).

The expression of *CacyBP/SIP* was also significantly upregulated in the striatum in YAC128 mice relative to control mice. This result was confirmed by western blotting, which showed an increase in a 30-kDa calcyclin (S100A6)-binding protein in all six studied samples. The CacyBP/SIP protein may play a role in the organization of microtubules and in calcium-dependent ubiquitination of target proteins (Filipek and Kuznicki, 1998; Matsuzawa and Reed, 2001; Filipek et al., 2002, 2008; Jurewicz et al., 2013).

The statistically significant upregulation of *Cib1* mRNA in YAC128 mice is consistent with a seven-fold increase in this gene's mRNA in microarray studies of the striatum in HD patients (Hodges et al., 2006). However, at the protein level this cannot be confirmed (Figure 2E), since we observed downregulation of CIB1 in samples from the striatum of five animals (0.15, 0.7, 0.85, 0.78, and 0.5-fold) and an increase in one (1.6-fold). We detected a statistically significant increase in *Cib2* mRNA and this result was confirmed by six independent western blotting experiments, showing an increase in CIB2 protein (Figure 2D). CIBs are EF-hand Ca^2+^ binding proteins identified in the brain (Bernstein et al., 2005; Blazejczyk et al., 2009). CIB1 has been implicated in neurodegenerative processes in AD, in which it was shown to interact with presenilin 2, but not presenilin 1 (Blazejczyk et al., 2006), but the role of CIBs in HD has not yet been confirmed.

Another gene that was significantly upregulated on the mRNA level in the striatum of YAC128 mice compared with control mice was *Calr*, however, the microarray studies did not detect upregulation of *Calr* in brain structures in HD patients (Hodges et al., 2006). Calreticulin is a multifunctional protein that acts as a transcription factor in the nucleus or binds Ca^2+^ in the ER (Michalak et al., 2009).

We detected statistically significant changes in the gene expression of *Psen2* mRNA in YAC128 mice and other components of γ-secretase complex, such as *Psenen* and *Aph1* isoforms. Considering that only the *Aph1b* gene met the criterion for Bonferroni correction, we speculate that this gene may by involved in the pathogenesis of HD. APH1 encoded by the *Aph1* gene is required for proteolytic activity and binds to the γ-secretase complex (Lee et al., 2004). Presenilins and Psenen are involved in the cleavage of the Notch receptor and APP processing (Francis et al., 2002; Goutte et al., 2002; Luo et al., 2003). Moreover, PSENs participate also in the modulation of Ca^2+^ signaling in the ER (Leissring et al., 2000; Supnet and Bezprozvanny, 2011). Thus, one can associate the altered gene expression of γ-secretase components with abnormal proteolytic activity in HD (Tarlac and Storey, 2003). It was suggested that HTT-interacting protein HIP1 (huntingtin-interacting protein 1) may provide a functional link between non-canonical Notch signaling-mediated neurogenesis through a *deltex*-dependent pathway (Moores et al., 2008).

In our experiments, most of the genes exhibited upregulation, in contrast to the results obtained from Affymetrix oligonucleotide array studies in HD R6/2 mice (Desplats et al., 2006), in which a decrease in the expression level of a majority of the genes was observed. R6/2 mice express exon 1 of human HD gene carrying 116 CAG repeats and exhibit a more progressive neurological phenotype (Mangiarini et al., 1996). However, comparing the results of high-throughput studies with our present study, which focused on a small group of genes, we identified some similarities. We and Desplats et al. (2006) detected a decrease in the expression of the *Rgs4* gene that encodes a protein involved in the regulation of G-protein signaling. Similarly, statistically significant downregulation of *Rgs4* was found in the striatum and motor cortex in HD patients using Affymetrix GeneChip microarrays (Hodges et al., 2006). The RGS4 protein inhibits G_*q*_, which, in turn, attenuates intracellular calcium signaling *via* phospholipase C (Berman et al., 1996). This is in line with results suggesting a cytosolic and mitochondrial Ca^2+^ overload in HD (Bezprozvanny and Hayden, 2004).

Cellular models of HD are another tool to detect the early, direct effects of mutant HTT without the influence of other secondary processes in advanced stages of the disease. We used PC12 cells with inducible expression of mutant HTT. Others have characterized this model in regard to HTT aggregates: 1 day after induction, only a few cells that expressed mutant HTT contained aggregates, whereas 5 days after induction, nearly all of the cells contained visible aggregates (Cong et al., 2005). We analyzed the cells 2 days after induction of HTT expression, which exhibited overexpression of expanded HTT protein (Figure 3A) and unchanged level of actin protein (Figure 3B) as found by immunoblotting. We observed a statistically significant reduction of calcium influx during SOCE, but not calcium content in the ER, in inducible huntingtin-expressing PC12 cells compared with control uninduced cells. This indicates that despite the likely association of mutant HTT with the ER (Rockabrand et al., 2007), the Ca^2+^ content of PC12 cells is not changed. Reduced activity of the SOCE in induced PC12 cells might be explained by our finding that *Orai2, Sept4* and *Calm3* are downregulated. The Orai calcium channel is activated during SOCE (Frischauf et al., 2008). Septin 4 has been shown to facilitate interactions between STIM1 and Orai1 proteins (Sharma et al., 2013). Microarray studies of the caudate from HD patients detected a seven-fold decrease in the expression of *Calm3* (Hodges et al., 2006). In induced PC12 cells, a decrease in the expression of another isoform of calmodulin, *Calm1*, has been described (van Roon-Mom et al., 2008). Calmodulins are multifunctional intermediate messenger proteins that transduce calcium signals by binding calcium ions and then modifying their interactions with various target proteins (Chin and Means, 2000). A previous study found that HTT interacts with calmodulin and that the expansion of polyglutamine altered this interaction (Bao et al., 1996).

In contrast to the observed decrease in SOCE in PC12 cells (this work) in cultured MSNs from YAC128 mice, an increase in calcium influx during SOCE was reported by Wu and coworkers. They demonstrated a key role of TRPC1 channels in supporting the SOC pathway in HD neurons (Wu et al., 2011). However, we did not find any differences in *Trpc1* gene expression in induced PC12 cells. The differences in SOCE dysregulation observed between the YAC128 mouse model by Wu et al. and our observation in PC12 cells may result from the length of mutated HTT, which can selectively affect gene expression. Alternatively, the difference could be due to the length of time mutated HTT was expressed. MSNs from YAC128 mice used by Wu et al. were cultured from 10–14 days, while PC12 cells were analyzed 2 days after HTT induction.

Although inducible PC12 cells and transgenic YAC128 mice show different changes in calcium influx during SOCE, each of these models has characteristic features of HD, such as the deposition of HTT aggregates mainly in the nuclei of inducible PC12 cells (van Roon-Mom et al., 2008) and nuclei of MSNs in the striatum in YAC128 mice (Van Raamsdonk et al., 2007). In both models, some similarities with the results obtained from samples from HD patients as well as other HD transgenic and cellular models were observed. In our experiments, the expression of *Rgs4, Hap1, Cib1*, and *Calm3* changed in the same direction as the changes reported by other studies.

This work presents for the first time convincing data demonstrating an increased expression of huntingtin-associated protein 1 in the striatum of HD mouse model at the mRNA and protein level. Our data showing upregulation of HAP1 are in line with those of Wu et al. (2011) when the observations of Tang et al. are taken into account (Tang et al., 2003, 2004). They found that mutant Htt *via* the interaction with HAP1 can activate IP_3_R1 *in vivo*. Thus, activation of SOCE reported by Wu et al. (2011) could be explained by the increased release of Ca^2+^ from ER due to the facilitated opening of the IP_3_R1. Since the dysregulation of neuronal Ca^2+^ represents an early event in the pathogenesis of models of HD (Zeron et al., 2002; Tang et al., 2003), the HAP1 protein might be considered as a potential therapeutic target in HD.

### Conflict of interest statement

The authors declare that the research was conducted in the absence of any commercial or financial relationships that could be construed as a potential conflict of interest.
